# Dynamics of plasma reconfiguration after pellet injection in Heliotron J

**DOI:** 10.1038/s41598-025-00993-5

**Published:** 2025-05-13

**Authors:** K. Ogihara, S. Inagaki, S. Kado, R. Matsutani, G. Motojima, S. Kobayashi, F. Kin, S. Ohshima, T. Minami, S. Konoshima, T. Mizuuchi, H. Okada, K. Nagasaki

**Affiliations:** 1 Graduate School of Energy Science, Kyoto University, Uji, Kyoto, 611-0011 Japan; 2 Institute of Advanced Energy, Kyoto University, Uji, Kyoto, 611-0011 Japan; 3 National Institute for Fusion Science, Toki City, Gifu, 509-5292 Japan; 4 University of California, Irvine, CA, US USA

**Keywords:** Heliotron J, Pellet injection, Reheat mode, Energy science and technology, Physics

## Abstract

Because of the many degrees of freedom in magnetized plasmas, the plasma is largely reconfigured when the plasma achieves a non-equilibrium steady state in response to changes in the internal environment. Here we report observations of a plasma reconfiguration process when the density is increased by pellet injection in Heliotron J. The plasma shows no adiabatic response to pellet injection and achieves an improved confinement during the reconfiguration process. It was found that plasma shrinking and expansion arose and the magnetic field line structure in the divertor region also changed in the plasma reconfiguration process.

## Introduction

Pellet injection, in which icy fuel pellets are injected into the plasma at high velocity, is the most promising method of fueling fusion plasmas. When the pellet ablation process is adiabatic, the stored energy in the plasma is almost unchanged. However, the stored energy has been observed to vary. On time scales longer than that of pellet ablation, the plasma is reconfigured through an increase in density and a decrease in temperature. During this reconfiguration phase, transitions to various confinement improvement modes have been observed in both tokamaks and stellarator/heliotron devices^1–7^. In particular, in stellarator/helliotron devices, the formation of internal diffusion barriers (IDBs)^8^ and reheat mode^9^ have been reported. Although not pellet injection, peaked electron temperature (*T*_e_) profile formation has also been observed in the reconfiguration phase after high-intensity gas-puffing^10^. These observations suggest that for the same pressure gradient, confinement varies depending on whether the density gradient or the temperature gradient is dominant. Confinement improvement in the density gradient dominant case may be explained by the stabilization of the temperature gradient-driven modes. In the W7-X study, the improvement in confinement is generally attributed to the suppression of ion temperature gradient (ITG) modes by the peaked density profile^11^. However, if turbulence is suppressed, the inward pinch necessary to form and sustain the peaked density profile is weakened, causing the plasma to eventually return to its pre-injection state. In addition to the reconfiguration of the plasma profile, the magnetic field configuration may also be reconfigured. In some tokamak devices, it has been observed that the steep density gradient generated by pellet injection drives a strong bootstrap current, leading to the formation of a reversed magnetic shear configuration^1^. In LHD, the connection between H-mode-like transitions and changes in the magnetic field structure at the edge has been discussed^12,13^. Thus, although it is widely recognized that pellet injection reconfigures plasma profiles, it is not obvious what the final profiles will be because energy and particle channels are coupled and there is feedback between the pressure profile and the magnetic field structure. Understanding the nature of such plasma is essential for achieving and maintaining high-temperature, high-density plasmas in magnetic confinement fusion. The purpose of this study is to clarify how the high-density plasma is reconfigured after pellet injection in the Heliotron J experiment.

## Reheat-like mode in Heliotron J

**Fig. 1:**
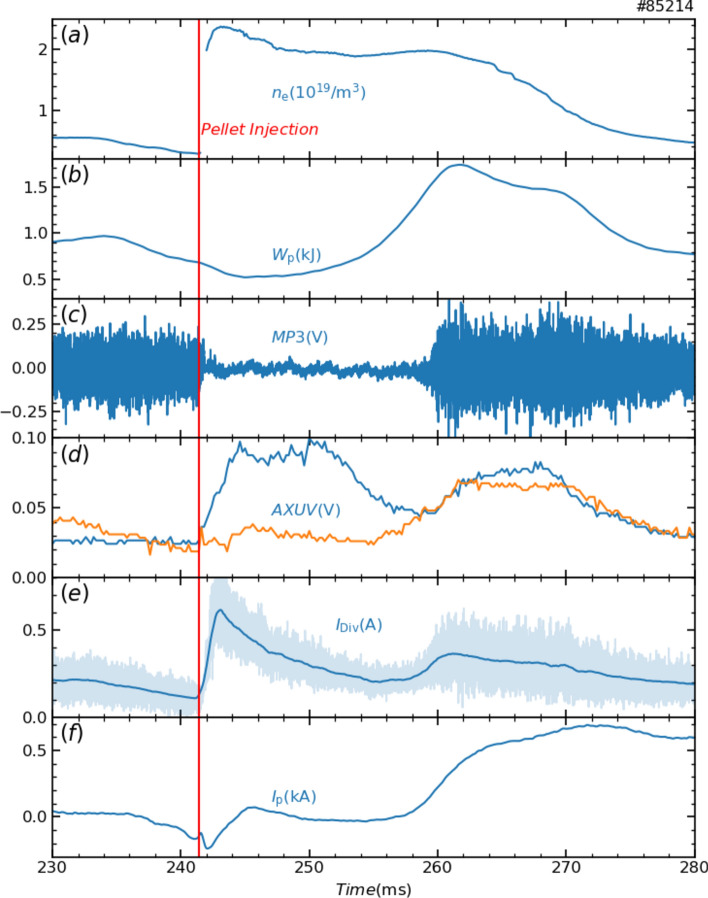
Typical temporal evolution of pellet discharge: (**a**) averaged electron density, (**b**) stored energy, (**c**) magnetic fluctuation, (**d**) line integrated radiation measured by AXUV detectors (blue and orange lines denote signals of the channels viewing the center and peripheral lines of sight, respectively), (**e**) ion saturation current of divertor probe, (**f**) plasma current. A pellet was injected at 241.4 ms.

Heliotron J is a helical-axis heliotron with a major radius of 1.2 m, an averaged plasma radius of *a* = 0.17 m, a plasma volume of 0.68 m^3^, and a magnetic field strength on the axis of 1.28 T^14,15^. Figure 1 shows the temporal evolution of the typical parameters with pellet injection in Heliotron J. The target plasma was produced with electron cyclotron resonant heating (ECRH) of 0.243 MW at the plasma center (70 GHz, 2^nd^ X-mode heating). The working gas was deuterium. At *t* = 241.4 ms, a hydrogen pellet was injected at a speed of 200–300 m/s^16^. The injected pellet was cylindrical, with a diameter of 0.7 mm and a length of 0.8 mm, and contained 1.6 × 10^19^ hydrogen atoms. The pellet penetrated close to the magnetic axis, supplying cold electrons and hydrogen ions through the ablation process. The expected increase in the averaged density, based on the number of atoms in the pellet and the plasma volume, is 2.3 × 10^19^ m^−3^. The averaged electron density, ${\overline{n} }_{\text{e}}$, was measured with a micro-wave interferometer (130 GHz) and fractional error is less than 1%, if the noise level is estimated from the root-mean-square value of the ${\overline{n} }_{\text{e}}$ in the absence of plasma. Pellet fueling caused the ${\overline{n} }_{\text{e}}$ to rapidly increase from 0.2 × 10^19^ m^−3^ to 2.4 × 10^19^ m^−3^ as shown in Fig. 1(a), which is roughly consistent with the expected increase (here the instantaneous ${\overline{n} }_{\text{e}}$ during ablation could not be traced due to fast fringe jump problem^17^. On the other hand, the stored energy, *W*_p_, measured with a diamagnetic loop, decreased (Fig. 1(b)). This decrease is partly due to increased radiation losses and partly due to the formation of a peak density profile, as measured with a Thomson scattering system^18^, where the plasma center exceeds the cutoff density (3 × 10^19^ m^−3^), which limits ECH absorption and lowers the absorbed power. We observed that the *W*_p_ increased abruptly 10 ms after the pellet ablation was completed. This phenomenon closely resembles reheat mode observed in CHS and other stellarator/heliotron devices in terms of the recovery of *W*_p_, the electron density peaking, and the increase in electron temperature in the periphery region^19–21^. Reheat is considered to be a common phenomenon in pellet-injected plasmas. Reheat mode has also been observed in supersonic molecular-beam injection (SMBI) discharges in Heliotron J^22,23^. However, the mechanism remains unclear.

**Fig. 2:**
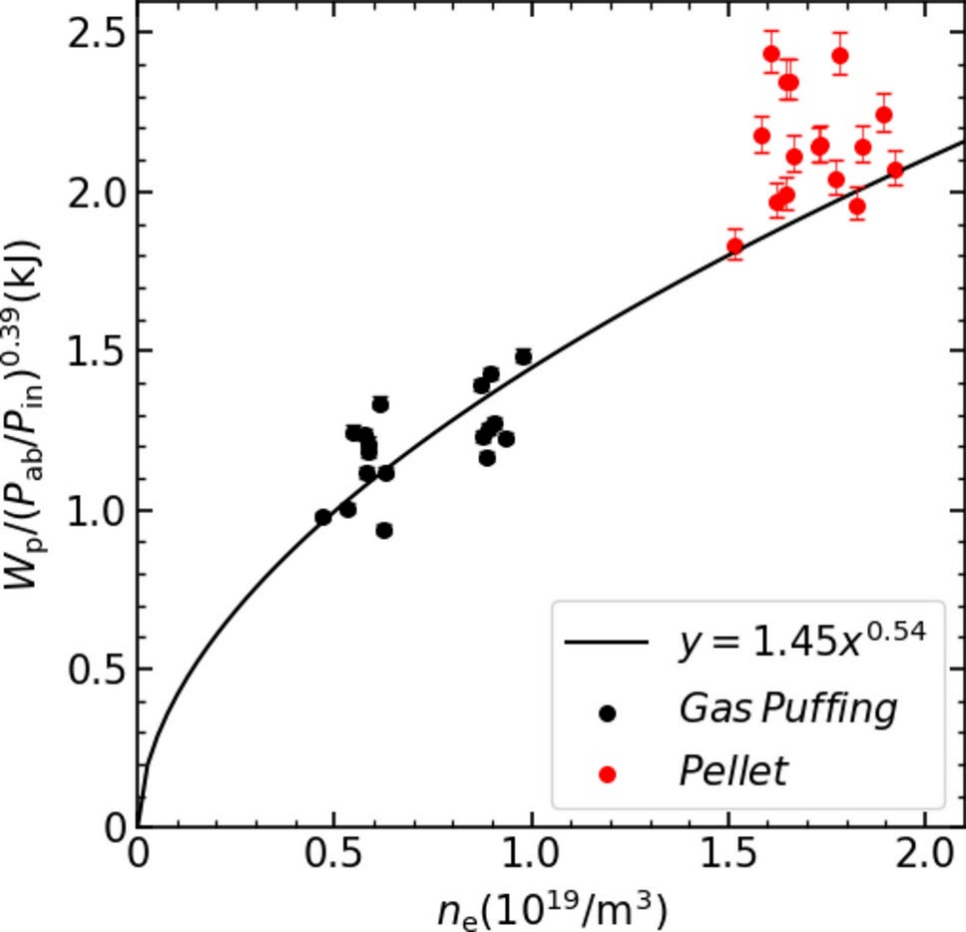
Comparison of the dependence of the maximum value of stored energy achieved in pellet and gas puffing discharge on average electron density.

**Fig. 3:**
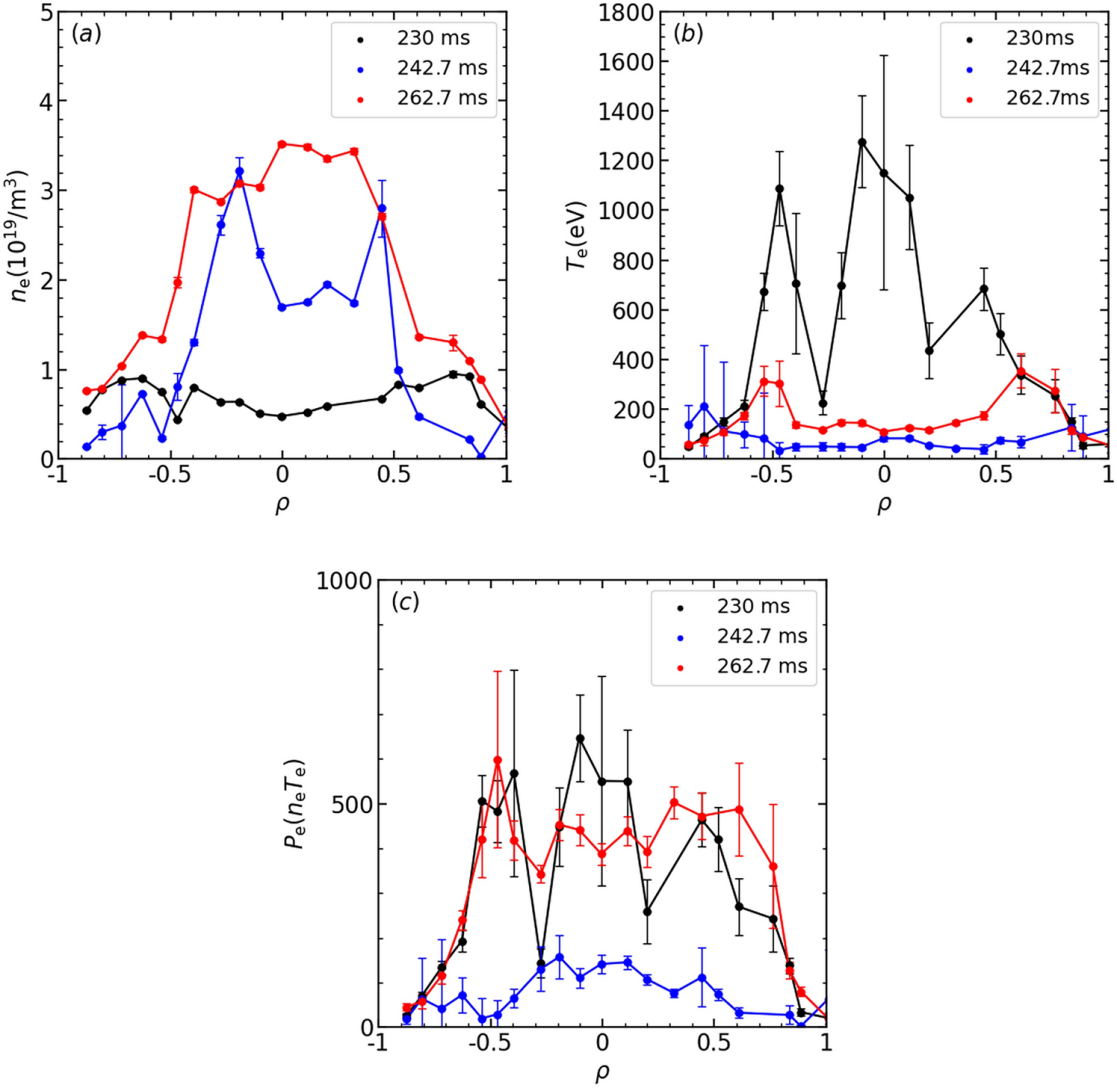
Radial profiles of (**a**) *n*_e_, (**b**) *T*_e_ and (**c**) *P*_e_ measured by tomson scattering at three different time-slices (230 ms (black), 242.7 ms (blue) and 262.7 ms (red)).

The stored energy reached its maximum during this reheat-like phase. Figure 2 compares the maximum ${W}_{\text{p}}$ in the reheat-like phase with the predicted density dependence of the maximum ${W}_{\text{p}}$ achieved in gas-puff operation, calculated using the density dependence of the ISS04 scaling^24^, i.e., ${W}_{\text{p}}={\tau }_{\text{E}}{P}_{\text{abs}}\propto {\overline{n} }_{e}^{0.54}{P}_{\text{abs}}^{0.39}$, here absorption power, ${P}_{\text{abs}}$, dependence is also considered. The absorption efficiency of ECH was calculated using TRAVIS code^25,26^ with the measured profiles of *T*_e_ and *n*_e_. The results indicate that the absorption efficiency of ECH was approximately 82% in gas-puff discharges, whereas it decreased to approximately 52% in pellet discharges. This reduction is due to ECH reflection in the cut-off density region. The solid line in Fig. 2 represents the ISS04 dependence (${W}_{\text{p}}{P}_{\text{abs}}^{-0.39}=c{\overline{n} }_{e}^{0.54}$), where the absolute value (proportional coefficient, *c*) was determined from the data set obtained in the gas-puff experiment. Since the reheat-like data points lie above this line, the confinement in the reheat-like mode is slightly improved compared to the standard gas-puff plasma. A significant local improvement could occur, as off-axis heating is not advantageous for increasing the stored energy. Even in ECH plasmas with additional NBI, pellet injection has a higher confinement performance than gas puff injection at the same density^27^. Figure 3 indicates radial profiles of* n*_e_, *T*_e_ and electron pressure (*n*_e_*T*_e_) at three time slices. (Variation of values in the *T*_e_ profile at 230 ms were large. An error-weighted multiple lookup table method was used to evaluate the *T*_e_ and error bars of the *T*_e_^28^. The target plasma density at 230 ms was close to the lower density limit when designing the measurement system (~ 0.5 × 10^19^ m^−3^)^18^. Even if the error bars are small, temperature measurement at low density thus seems to have a larger error due to low scattered light in this experiment). Immediately after pellet ablation (*t* = 242.7 ms), the density increased while the temperature decreased. This process was non-adiabatic, as the electron pressure dropped to less than half its pre-injection level (*t* = 230 ms). However, the stored energy did not show a significant decrease, suggesting that the energy had transferred from electrons to ions due to the increase in collisions. When the stored energy was recovered and further increased in the reheat-like phase (*t* = 262.7 ms), the electron pressure recovered to pre-injection level, despite being over dense plasma, i.e. no central heating. The further increase of the stored energy in the reheat-like phase suggests a greater contribution from ion pressure. In fact, significant increases in ion temperature after pellet injection have been observed in other devices^29,30^.

The dynamic behavior of the plasma after pellet injection cannot be explained only by the transition to reheat mode. Magnetic fluctuations measured with a magnetic probe were suppressed immediately after pellet injection due to the shrinking of plasma, here shrinking means that the electron pressure decreased in the edge region and the plasma volume appears to have decreased as shown in Fig. 3(c) at *t* = 242.7 ms. Subsequently, *W*_p_ began to rise (onset of the reheat-like phase). The onset of re-excitation of the magnetic fluctuations was delayed from the onset of the reheat-like phase. The re-excitation of magnetic fluctuations may be caused by an increase in the peripheral pressure gradient, i.e. plasma expansion. The magnetic fluctuations increased with *W*_p_ and then saturated, followed by a delay of about 1 ms, after which the *W*_p_ saturated (onset of the saturation phase) as shown in Fig. 1. The power spectrum of magnetic fluctuations in the saturated phase extended over a bandwidth of several 10 kHz and no coherent modes were observed. We consider that the input power and loss driven by broad magnetic fluctuations were balanced, hence the peripheral pressure gradient saturated, and then the *W*_p_ also saturated in the saturation phase. During the transitions from the shrinking phase to the expansion phase, and then to the saturation phase, the plasma profile and magnetic field structure were both observed to be reconfigured. Signals of the AXUV array and the divertor probe array were synchronized with these reconfigurations (details are provided in the next section). Plasma current measured with the Rogowski coil indicates an impulse-like response just after pellet injection, however the change in *W*_p_ at that time was less than 10%, and no significant effect was observed. Thus, plasma currents would not play an important role in the shrinking and expansion processes.

## Plasma shrinking and re-expansion after pellet ablation

**Fig. 4:**
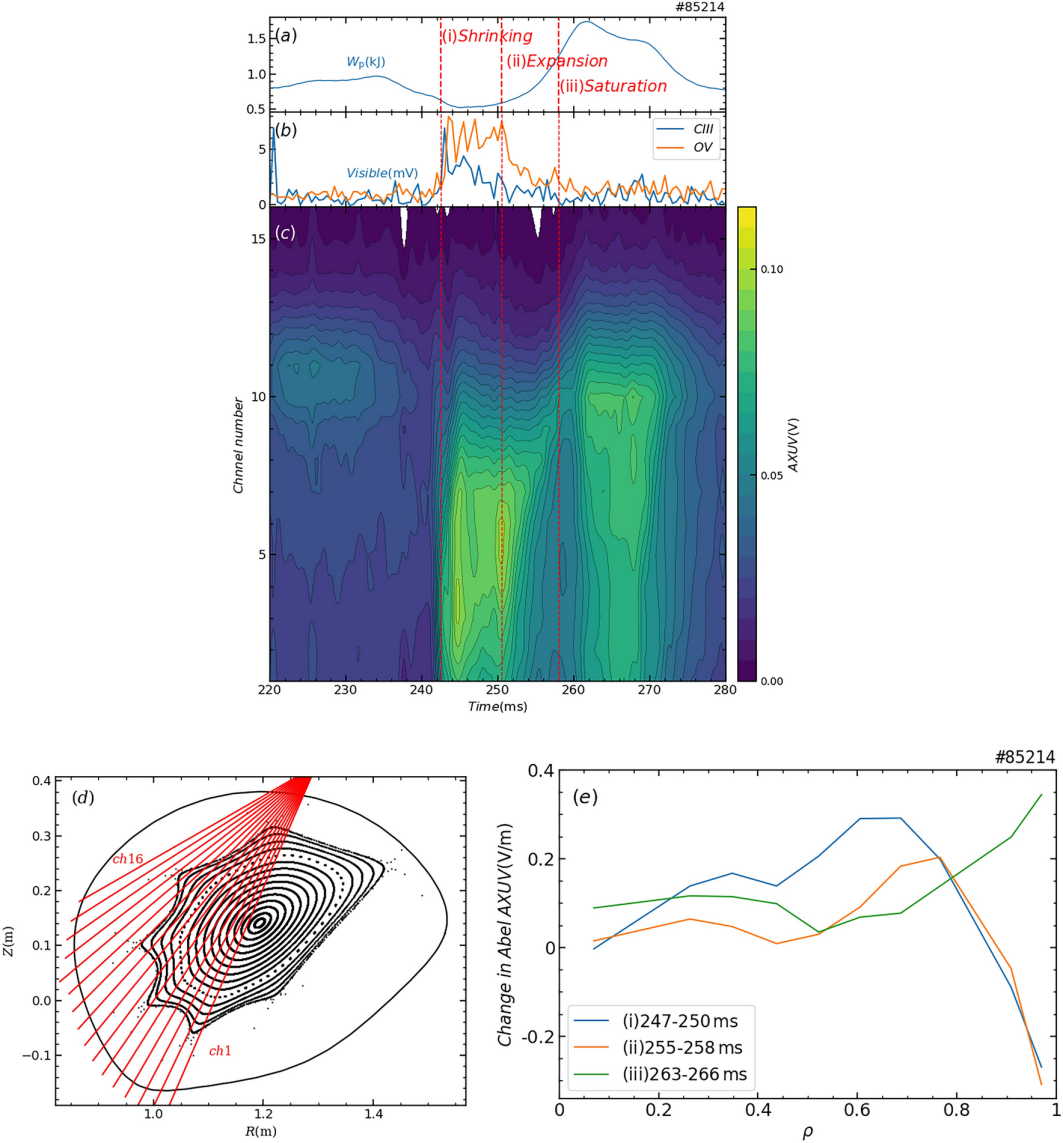
Reconfiguration of the spatial structure of radiation in three different phases. Temporal evolutions of (**a**) stored energy ((i), (ii), and (iii) denote shrinking phase, expansion phase, and saturation phase, respectively), (**b**) impurity line-intensities (blue and orange lines denote CIII: 464.7 nm and OV: 278.1 nm, respectively). (**c**) Contour plot of line integrated radiation measured by an AXUV array. (**d**) Magnetic flux surfaces and viewing cords of the AXUV array. (**e**) Difference of the Abel-inverted radiation profile from the steady state (220–230 ms) in three different phases.

The reconfiguration process of the spatial structure of plasma was traced using a 16-channel AXUV photodiode array, which detects line-integrated radiation from the plasma across a wavelength range of 0.0124–1100 nm. Figures 4(a) and (b) show the time evolution of *W*_p_ and the line intensities of CIII and OV, respectively. The three phases (shrinking phase, expansion phase, and saturation phase) are separated by three vertical red dashed lines. Figure 4(c) presents a contour map of the AXUV signals, while the relationship between the vacuum magnetic surface and the line of sight of the AXUV array is shown in Fig. 4(d). In this arrangement, smaller channel numbers correspond to viewing chords closer to the plasma center. Before pellet injection, the radiation was relatively weak over the entire plasma region because of its low density, and the peripheral region exhibited slightly higher radiation intensity than the center, suggesting that the temperature in the peripheral region is lower and the impurity radiation intensity is relatively large. Carbon and oxygen are the principal impurities in Heliotron J plasmas. After pellet injection, carbon and oxygen in the central region recombined to lower ionization states through a significant decrease in central temperature and a significant increase in central density and thus the line intensities of CIII and OV increased in the shrinking phase. Although the AXUV signal from the central channel also increased, one from the peripheral region did not increase as shown in Fig. 1(d). These observations indicate that *P*_e_ in the peripheral region decreased due to a decrease in *n*_e_ as shown in Fig. 3(a), i.e., plasma was shrunk. This shrinking phase persisted for approximately 8 ms (phase (i) in Fig. 4(a)), after which the plasma abruptly began to reconfigure its radiation structure. The radiation peak shifted toward the periphery, line intensities of OV and CIII began to decrease and *W*_p_ started to rise. These trends indicate that the central temperature increased (re-heated) through the increase in the *P*_e_ in the peripheral region, i.e. expansion of the plasma. This expansion phase lasted for another 8 ms (phase (ii) in Fig. 4(a)) and was then terminated by a sudden onset of magnetic fluctuations. Following the end of the expansion phase, the *W*_p_ and magnetic fluctuations were saturated, and maximum *W*_p_ was achieved in this saturation phase (phase (iii) in Fig. 4(a)). Although the detailed mechanism of the reheat-like mode has not been clarified, there may be a competing process of plasma expansion and its suppression by magnetic fluctuations. The onset of the reheat-like mode has also not been apparent in the temporal evolution of *W*_p_, but it was clear that the radiation structure reconfigured fast in each phase as shown in Fig. 4(c). The difference of the Abel-inverted radiation profile from the steady state (220–230 ms) indicated that the radiation profile in the core region changed from shrinking to expansion and the radiation was localized in the edge region in the saturation phase (Fig. 4(e)). As a result of the reconfiguration, the radiation profile was significantly different between before pellet injection and in the saturation phase.

## Changes in divertor structure

**Fig. 5:**
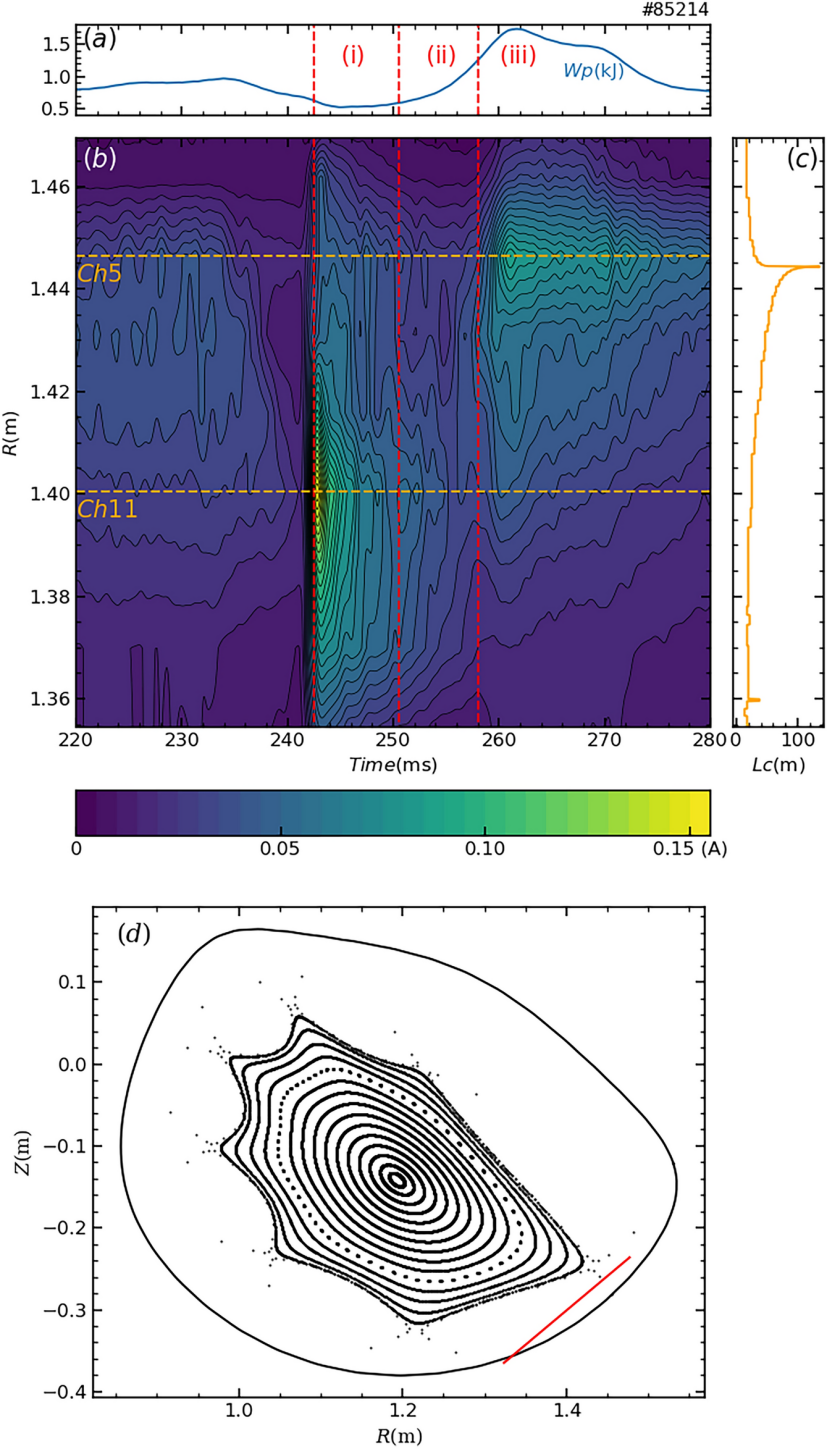
Changes in the distribution of divertor flux. (**a**) stored energy (**b**) contour graph for divertor prove array (**c**) the length of the magnetic field lines passing through the measurement points (**d**) positions of vacuum magnetic surface and divertor plate.

The magnetic field structure is also reconfigured in conjunction with the reconfiguration of the spatial structure of the plasma. Changes in the magnetic field structure in the scrape-off layer (SOL) region were observed by the footprint of the magnetic field lines on a divertor plate, identified through the movement of the ion saturation current peak. Figure 5(a) shows the temporal evolution of *W*_p_ and the duration of each phase. Figure 5(b) illustrates the spatiotemporal behaviour of the time-averaged ion saturation current (with a 1 ms averaging time window) on the divertor plate, measured with a 9-channel probe array (nine of the 20 channels were used in this experiment). Figure 5(c) presents the length of the magnetic field line intersecting each probe tip, as calculated using KMAG code^31^. The short field line length implies that the magnetic field lines immediately intersect the wall, whereas the long field lines indicate that they reach the vicinity of the last closed flux surface. Therefore, the ion saturation current is expected to be large at probe tips with longer field line lengths. The spatial relationship between closed flux surfaces and the divertor plate is shown in Fig. 5(d). In the saturation phase (phase (iii) in Fig. 5(a)), the ion saturation current is maximum near the location where the magnetic field line length is the longest. This tendency was also observed prior to pellet injection. However, in the shrinking phase (phase (i) in Fig. 5(a)), the peak of ion saturation current distribution did not correspond to the location of the longest magnetic field line length, but rather matched to the location where ion saturation peak appeared in the plasma initiation phase. This positional difference in the peak of ion saturation current suggests a change in the magnetic field structure. The peak of the ion saturation current rapidly moved about 5 cm on the divertor plate. Since such a shift would typically require a plasma current exceeding 2.0 kA^32^, this change in the magnetic field structure cannot be explained by the observed plasma current. Notably, while the radiation structure changed gradually during the expansion phase, the shift in divertor structure occurred abruptly. This implies that plasma expansion preceded the reconfiguration of the magnetic field structure.

**Fig. 6:**
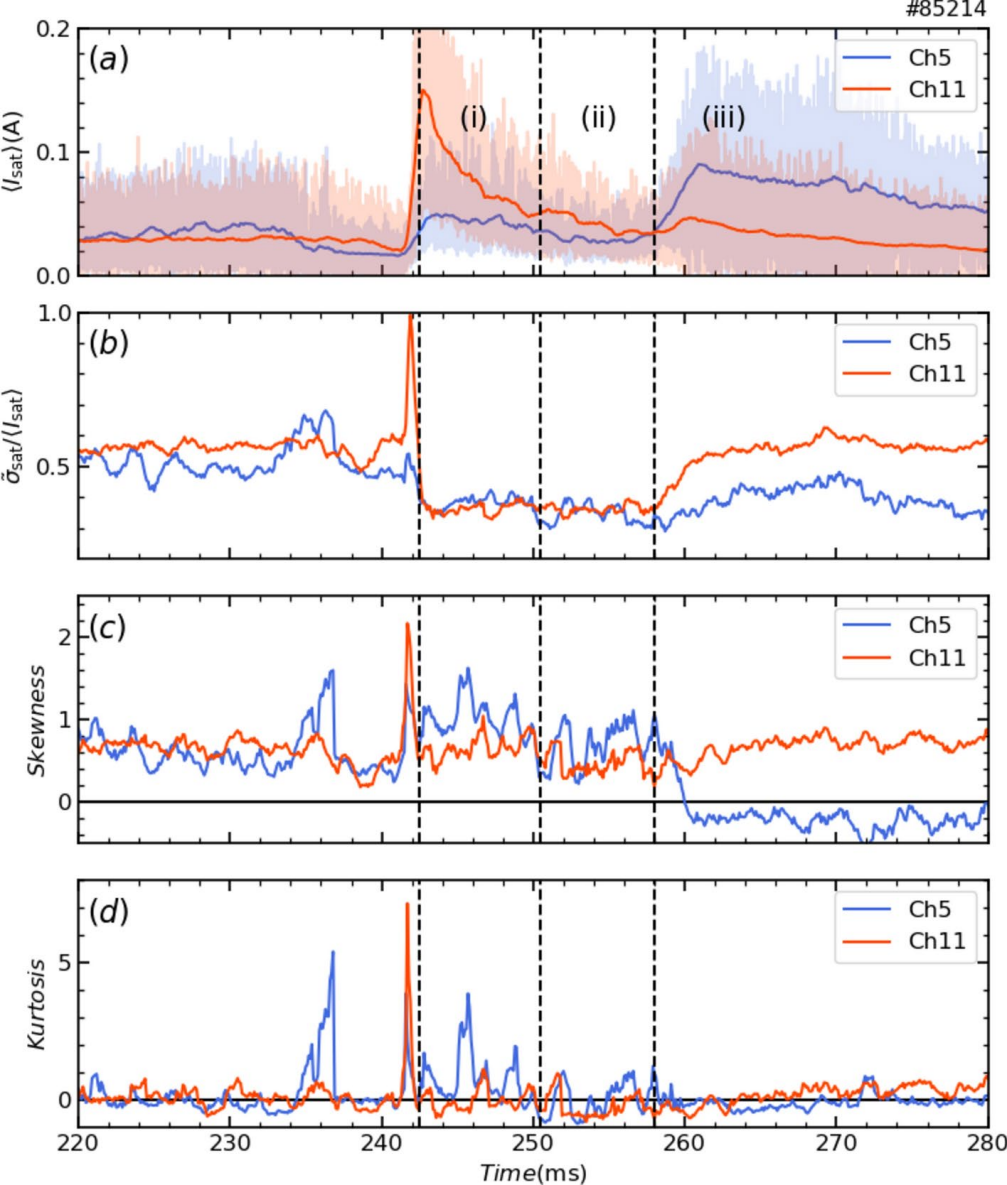
Time variation of (**a**) divertor probe signals and the time-averaged value in the peak channels, their (**b**) normalized fluctuation levels, (**c**) skewness and (**d**) kurtosis. Raw signals are indicated by lighter red and blue lines in (**a**).

Figure 6 shows the temporal evolution of the time-averaged value $\langle {I}_{\text{sat}}\rangle$, the normalized fluctuation level ${\widetilde{\sigma }}_{\text{sat}}/\langle {I}_{\text{sat}}\rangle$, skewness and kurtosis of the ion saturation current measured by probe tips of channel 5 and 11, which are located at peaks in the shrinking and saturation phases. Here, ${\widetilde{\sigma }}_{\text{sat}}$ denotes the standard deviation. Skewness and kurtosis are measures of asymmetry and sharpness, respectively, of the probability density function (PDF) of the ion saturation current signal. A time window of 0.5 ms was used to calculate the time evolution of these statistical measures. If the PDF is Gaussian, skewness and kurtosis are zero. The physics of random collision processes (Brownian motion) is thought to lie behind the Gaussian distribution. Since ion saturation current reflects particle flux along magnetic field lines, the PDF is expected to be Gaussian if the cross-field transport is diffusive. On the other hand, when plasma blobs come to the divertor probe, positive spikes are observed in the ion saturation current and it shows positive skewness^33^. The skewness and kurtosis of the ion saturation current were indeed nonzero. This suggests that the stochastic process generating the divertor flux is non-stationary (abrupt) and non-random diffusion. In addition, skewness and kurtosis were large in the shrinking phase and became small in the saturation phase. In particular, the skewness of channel 5 changed sign in the saturation phase. This indicates that transport in the SOL was qualitatively different for the shrinking phase and the saturation phase. The abrupt changes of skewness and kurtosis of channel 5 from the expansion phase to the saturation phase were as rapid as the transition.

## Discussion

In this study, we identified a reconfiguration process of spontaneous shrinkage and expansion of the plasma, accompanied by a change in the magnetic field structure in the SOL region after pellet injection. However, the causal relationship between the reconfiguration and the increase in *W*_p_ has not been clarified yet. Changes in the magnetic configuration in the edge region, as seen in LHD^34^, may contribute to the increase in *W*_p_. In fact, at the end of the expansion phase, the magnetic field structure back-transitioned abruptly, magnetic fluctuations increased and the rise in *W*_p_ stopped. At this back-transition of magnetic field structure, the sudden large change in total plasma current was expected because a 2 kA increase is required to explain the observed change in the magnetic field structure (i.e., the movement of the divertor striking point). However, such a change in plasma current was not observed. Even if the total plasma current did not change, the current profile may have changed significantly due to the increased peripheral resistance caused by pellet injection. Unfortunately, we have not been able to measure changes in the magnetic field structure in the core, which are necessary to verify changes in the current profile. With respect to turbulence, the electromagnetic fluctuations that disappear during shrinkage remain quiet during the expansion phase, and the level of fluctuations in the divertor probe is also lower during expansion. Turbulent transport appeared to be minimized in the expansion phase due to the reconfiguration of plasma profiles. Collisionality, which increased in the shrinking phase, gradually decreased as a result of the reconfiguration. Since collisionality contributes to both the excitation and suppression of turbulence and affects neoclassical transport, its role is complex. In plasmas where neoclassical transport is somewhat large and both collisional and turbulent transport coexist, an optimal level of collisionality may exist that minimizes total transport, as observed at LHD^35^. More detailed turbulence measurements are left for future work.

Observed changes in stored energy and average density over time in Heliotron J are similar to the thermal quench and reignition phenomena observed in the W7-X, LHD, TJ-II^36^. When the plasma state after reignition or reheat is the same as before pellet injection, it is indeed a reignition. In this experiment, the plasma state is different from that before the pellet injection (the plasma electron pressure profile is the same, but the density gradient is larger than the temperature gradient, and the radiation profile is also different) through the reconfiguration process. The difference between reignition and reconfiguration may be related to the fact that the plasma has not progressed to a complete thermal quench by radiative collapse in this experiment. This may also suggest that the time scale of the reconfiguration is shorter than the time scale of the thermal quench. The observed time scales of changes in the radiation profile and magnetic field structure in the divertor were shorter than the energy confinement time.

## Summary

In summary, the reconfiguration process of the plasma profile and magnetic field structure after pellet ablation was identified in the Heliotron J plasma. Experimental observations indicated:

(i) The plasma pressure spontaneously shrank and expanded, and the magnetic field line structure in the SOL/DIV region changed synchronously,
(ii) Reconfiguration accompanied a reheat-like improvement of energy confinement.
(iii) The reheat-like phase termination was synchronized with the end of the expansion phase and back-transition of the magnetic field structure.

The identification of spontaneous structural reconfiguration processes driven by pellet injection will contribute greatly to comprehensive understandings of the confinement improvement in the high-density plasmas and creating the optimized operation scenario for burning plasma.

## Data Availability

The datasets analysed during the current study are available from the corresponding author on reasonable request.
